# Molecular characterization of *Pasteurella multocida *isolates obtained from poultry, ruminant, cats and dogs using RAPD and REP-PCR analysis

**Published:** 2016-09

**Authors:** Hesamaddin Shirzad-Aski, Mohammad Tabatabaei

**Affiliations:** Department of Pathobiology, School of Veterinary Medicine, Shiraz University, Shiraz, Iran

**Keywords:** *Pasteurella multocida*, Molecular analysis, RAPD, REP-PCR

## Abstract

In the present study, Randomly Amplified Polymorphic DNA (RAPD) and Repetitive Extragenic Palindromic sequence-based Polymerase Chain Reaction (REP- PCR) were used to characterize 131 isolates of *Pasteurella multocida, *originating from different healthy and diseased animal species obtained from several geographical regions of Iran. The RAPD and REP-PCR generated amplified products in the range of 300 to 3400 bp and 200 to 2850 bp, respectively. Among all of the *P. multocida *isolates, cluster analysis revealed that 63 clusters and nine untypable isolates and 81 clusters and six untypable isolates were produced with RAPD and REP-PCR methods, respectively. The results indicated that the REP-PCR method showed a slightly higher level of discrimination power in differentiating of *P. multocida *isolates as compared with RAPD. The results showed that a considerable level of genetic diversity exists among *P. multocida *isolates even in the isolates with the same animal or geographical origins. There was no host- and region-specific pattern. In addition, the isolates obtained from the healthy and diseased animal did not reveal any correlation genotypic profiles, which could be supported by the hypothesis that *P. multocida *is a strictly opportunistic pathogen. In conclusion, because of a large amount of genetic heterogeneity in the *P. multocida *isolates, Pasteurellosis may be caused by different clones in the same herd or animal.

## INTRODUCTION

The Gram-negative bacterium, *P. multocida*, is an important worldwide primary and opportunistic pathogen as well as the common commensal of many wild and domesticated animals [1-3]. It is also associated with human infections following by a cat and dog bite or scratch [4]. *P. multocida *has a broad host range, but the source of pathogenic isolates responsible for most sporadic infections remains unclear [5]. Strains of *P. multocida *are divided into five capsular types (A, B, D, E and F), and investigators believe that each capsular type is nearly associated with a specific host or disease [6- 10].

Identification and differentiation of pathogenic bacteria strains are the two main requisites for the epidemiological studies. The phenotypic methods, like serotyping and biotyping, have been used to differentiate the strains, but these methods are so hard, extremely tedious and often produce unclear results. Thus, in the recent years, the phenotypic differentiation tools have been frequently replaced with the genotypic methods [1, 7, 9]. As compared to the phenotypic tools, the genotypic methods such as RAPD and REP-PCR provide more accurate results, offer more reproducibility, and also they save time [7, 11, 12].

The RAPD technique relies on the polymorphic DNA that can be amplified by one or several short oligonucleotide primers of the arbitrary sequences with 8–12 nucleotides. The profiles of DNA fragments among the strains produced by the RAPD primers can be compared and analyzed [13]. Since the RAPD is a simple, fast and sensitive method, it is one of the most promising genotyping techniques, which has been used to differentiate closely related bacterial species and strains [5, 9]. This method has been used for specific detection and differentiation of the *P. multocida *strains [14] and it has been shown that the results of this procedure are reproducible and reliable [9, 13, 14].

The REP elements are repetitive palindromic short sequences distributed in the extragenic DNA regions of bacteria like *Escherichia coli. *The specific primers for the REP-PCR complement these repetitive sequences and provide the reproducible and unique REP-PCR DNA fingerprint patterns [15,16]. In general, the REP-PCR method is a valuable tool for the rapid epidemiological analysis and characterization of bacteria and it has been used in several studies [17, 18, 19]. Additionally, it was employed for the molecular typing of the *P. multocida *strains [17, 20, 21, 22].

The main aim of the present study was to use the RAPD and REP-PCR assays for the characterization of various *P. multocida *isolates obtained from different hosts and geographic locations in Iran. Moreover, the efficiency of the RAPD and REP-PCR analysis was compared.

## MATERIALS AND METHODS

**Bacterial strains: **A total of 131 *P. multocida *isolates, obtained from apparently healthy and diseased animals, including bovine (11), sheep (44), goat (24), cats (21), dogs (16), and poultry (15), which were isolated between February and September 2014, were used in this study. The origins of these isolates were different regions of Iran. The isolates were obtained from external nares and/or pneumonic lungs of the bovine, sheep, and goat (Shiraz, Iran); oral cavity and tonsil of cats and dogs (Tehran and Shiraz, Iran). Moreover, the isolates were gained from blood or liver samples from suspected birds such as duck (north of Iran), chicken (north of Iran) and turkey (Tehran and Shiraz, Iran), and bovine (Varamin and Tehran, Iran) with septicemic symptoms. Isolates were previously confirmed as *P. multocida *via standard bacteriological methods and *P. multocida*-specific PCR [23] and stored at -80 °C. Each isolate was plated on the blood agar plate (Oxoid, United Kingdom) supplemented with 5% fresh sheep blood and incubated aerobically at 37 °C for 48 h. The *P. multocida *strain 85020 (wild type, type B: 2) was included as a positive control.

**DNA extraction: **The isolates were cultured overnight in the Brain-Heart Infusion (BHI) broth (Oxoid, United Kingdom) at 37°C and then were used for DNA extraction using a boiling procedure according to Ewers et al. [2] method. The purity and concentration of the DNA were estimated by spectrophotometry at 260 and 280 nm.

**The RAPD and REP-PCR fingerprinting: **The RAPD amplification was performed using two primers reported for the typing of *P. multocida*. The random OPA- 11 (5’-CAA TCG CCG T-3’) and AP2 (5’-GTT TCG CTC C-3’) primers were used to determine the genetic differences among the *P. multocida *isolates [24, 25]. The RAPD reaction was performed in a final volume of 25 μl consisting of 2 μl DNA template, 2.5 μl 10 X PCR buffer (75 mM Tris-HCl, pH 9.0, 2 mM MgCl2, 50 mM KCl, 20 mM [NH4]2SO4; CinnaGen, Iran), 1 μl deoxynucleoside triphosphates (dNTPs, 50 mM; CinnaGen, Iran), 1.25 μl (1.25 U) of Taq DNA polymerase (CinnaGen, Iran), and 1 μl (25 pmol) of each primer. The volume of the reaction mixture was reached to 25 μl using distilled deionized water. Negative control contained all elements except the template DNA. The Amplification reactions were carried out in a thermal cycler (MJ mini, BioRad, USA) using these cycling parameters: initial denaturation at 94°C for 5 min, followed by 45 cycles of denaturation at 94 °C for 1 min, annealing at 37°C for 1 min and extension at 72 °C for 3 min. A final step of the extension was applied at 72°C for 10 min and the PCR products remained in the thermal cycler at 4 °C until they were collected. One pair primers targeting REP sequences of *P. multocida*, (REP1R-1Dt 3’- CGG NCT CAN GCN GCN NNN-5’ and REP2-1Dt 5’-NCG NCT TAT CNG GCC

TAC-3’), were used for the typing of *P. multocida *as described previously by Versalovic *et al. *[26] and Saxena *et al. *[22]. Briefly, DNA was quantified and 50 ng of the genomic DNA was used in the 25 μl reaction mixture containing 200 μmol/L of each dNTP, 20 pmol of each primer, 1.5 mM of MgCl2 and 2 U of the Taq DNA polymerase in the 1 X PCR buffer. All reagents were supplied by CinnaGen, Iran. The PCR amplifications were performed with an initial cycle of denaturation (94°C for 5 min) followed by 30 cycles of the denaturation (94°C for 1 min), the annealing (42°C for 1 min) and the extension (72°C for 3 min), with a single final extension at 72°C for 10 min.

The RAPD and REP-PCR products were separated by electrophoresis on a 2% agarose gel at 10 V/cm in 0.5 X TBE (Tris-borate-Ethylenediaminetetraacetic acid) buffer. The bands were visualized after staining with ethidium bromide (0.5 μg/ml) and photographed under UV illumination (BTS-20, Japan). The 100-bp (CinnaGen, Iran) and One kb DNA ladders (MBI, Fermentas) were used as molecular size markers. The fingerprints were saved in a tagged image files (tiff) format for further analysis. Three independent PCR and electrophoresis techniques were done for each isolate to check the reproducibility of the both PCR reaction assays.

**The RAPD and REP-PCR data analysis: **The RAPD and REP-PCR banding patterns were analyzed using BioNumerics 7.5 software (Applied Maths, Belgium). The software was used to crop each image, remove the background, artifact, and frame lanes, adjust the gel distortion and do an initial identification of the bands. The sensitivity of the software was adjusted for each gel and each lane was manually curated as needed to ensure the consistency across gels. The strains were clustered according to the Dice coefficient by unweighted paired group method of arithmetic averages (UPGMA) and a 5% positional tolerance. The resulting dendrogram was partitioned at an 80% similarity cutoff to determine final groupings for the RAPD and REP-PCR methods and to identify and establish the relationship among various strains, species, and geographical area.

## RESULTS

Two RAPD primers, which were developed for the *P. multocida, *were used to evaluate their discrimination power for the tested isolates. There was a variation in the pattern of amplified fragments generated by each primer. For a suitable handling of data, it was decided to select the primer AP2 that generated few major bands. The REP- PCR fingerprinting was chosen as the second DNA fingerprinting method. The RAPD and REP-PCR produced bands ranging from approximately 300 to 3400 bp and 200 to 2850 bp, respectively. The number of polymorphic DNA fragments of *P. multocida *isolates obtained from RAPD and REP-PCR varied from one to seven and one to eight bands, respectively. The details of bands are shown in Table 1 and 2.

**Table 1:** The results of RAPD typing method for 131 *P.* *multocida* isolates

Animal species (number of isolates)	Number of bands	Range of bands (bp)	Number of clusters	Number of untypable isolates
Bovine (11)	3-5	300-3400	3	0
Sheep (44)	2-7	400-2800	9	4
Goat (24)	1-6	450-2800	9	0
Cat (21)	2-5	450-2950	7	1
Dog (16)	3-4	450-2700	5	1
Poultry (15)	2-7	350-2300	3	3

**Table 2:** The results of REP-PCR typing method for 131 *P. multocida* isolates

Animal species (number of isolates)	Number of bands	Range of bands (bp)	Number of clusters	Number of untypable isolates
Bovine (11)	3-5	450-2050	3	1
Sheep (44)	2-5	450-2800	18	3
Goat (24)	3-7	200-2700	11	1
Cat (21)	2-6	450-2600	9	1
Dog (16)	1-8	450-2850	8	0
Poultry (15)	3-6	400-2000	7	0

**Figure 1:**
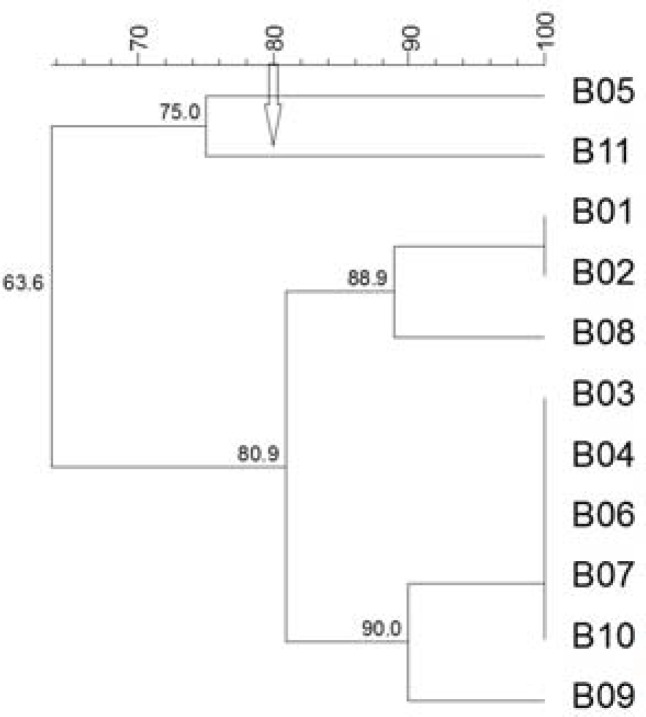
Dendrogram of bovine isolates of *P. multocida *obtained from the RAPD profiling using the UPGMA analysis. The values found in the groups indicate the percentage of similarity. The clusters were formed by 80% of similarity among the isolates. PM, *P. multocida*; B, Bovine

The patterns were analyzed by UPGMA according to 80% cutoff value. Genotypic characterization of *P. multocida *isolated from bovine using the RAPD analysis showed three major clusters (Fig. 1).

**Figure 2:**
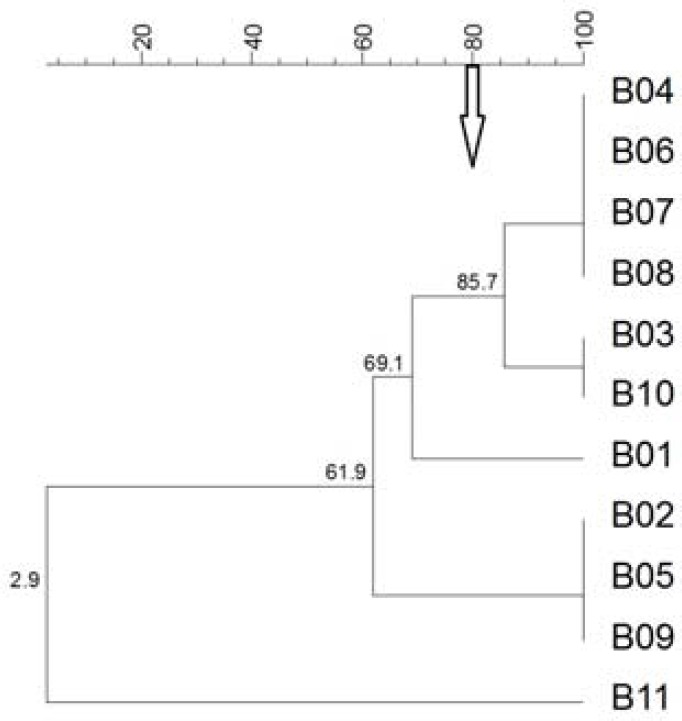
Dendrogram of bovine isolates of *P. multocida *generated from the REP-PCR patterns using the UPGMA analysis. The scales indicate the percentage of similarity. The arrow shows 80% cutoff value for differentiation. PM, *P. multocida*; B, Bovine

One group contained 9 isolates and two groups contained one isolate each. In addition, the group with nine isolates had two subclusters. On analyzing the same field isolates by REP-PCR, three main clusters were observed (Fig. 2).

**Figure 3:**
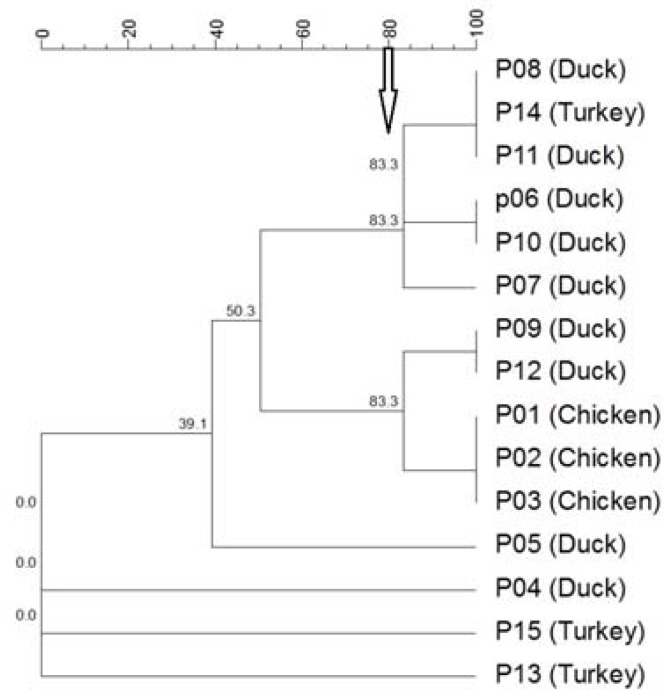
Dendrogram of avian isolates of *P. multocida *generated from the RAPD banding profiles using the UPGMA analysis. The values found in the groups indicate the percentage of similarity. The arrow indicates the cutoff value (80%) for cluster analysis. PM, *P. multocida*; P, Poultry

The largest group contained six isolates with two subclusters, one group contained three isolates and the remaining group contained one isolate. Moreover, one strain was untypable with this method. The number of the groups that obtained from other animals are shown in Tables 1 and 2. Briefly, on computational analysis by RAPD, the isolates obtained from the sheep, goat, dogs, cats and poultry had nine clusters and four isolates without any bands, nine clusters, seven clusters and one untypable isolate, five clusters and one untypable isolate, and three clusters and three isolates without any bands, respectively (Fig. 3).

**Figure 4:**
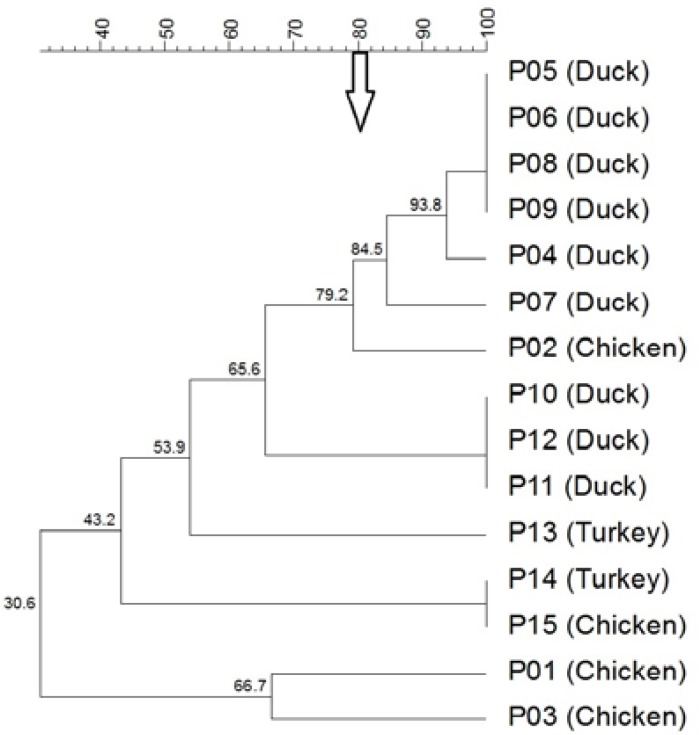
Dendrogram of avian isolates of *P. multocida *generated from the REP-PCR profiling using the UPGMA analysis. The values found in the groups indicate the percentage of similarity. The arrow shows 80% cutoff value for differentiation. PM, *P. multocida*; P, Poultry

Based on the REP-PCR clustering in the isolates obtained from the sheep, goat, dogs, cats and poultry, 18 clusters and three untypable isolates, 11 clusters and one untypable isolate, nine clusters and one untypable isolate, eight clusters and seven clusters were observed (Fig. 4). In addition, the subclusters were also observed for some main clusters, which reflected a good discrimination capability of both techniques. The

*P. multocida *isolates produced 63 clusters and nine untypable isolates with RAPD method and 81 clusters and six untypable isolates with REP-PCR method. A slightly higher level of discrimination power was observed using the REP-PCR method for the characterization of *P. multocida *isolates, as compared to the RAPD method. Since the isolates from the different animals were distributed in all the cluster groups, there was no specificity that could be designated the host specificity. Similarly, there was no specific clustering of isolates depending on their geographic properties, as the isolates with different geographic origin exhibited cross-clustering. In addition, the absence of any significant correlation between the isolates from healthy and diseased animals with different anatomical location was noticed in both PCR typing methods.

## Discussion

Since the RAPD and REP-PCR typing methods have previously been shown to provide the sufficient discrimination of *Pasteurella *isolates [9, 18, 20], these methods were used in this study to determine the characterization of *P. multocida *isolates obtained from different sources. The results demonstrate that the RAPD assay has a sufficient discrimination power for *P. multocida *typing. In addition, this method shows that the high genetic heterogeneity exists among the *P. multocida *isolates.

In agreement with the results of this study, Dziva *et al. *[24] and Lee *et al. *[27] reported a relatively large amount of the genetic heterogeneity in the *P. multocida *isolates. Moreover, similar to the results of this research, Dziva *et al. *[24] showed that the RAPD method with the similar primer produced one to six bands in a range between 300 and 2000 bp. Lee *et al. *[27] obtained 16 different clusters from 69 strains of *P. multocida *with the RAPD profiles. However, in contrast to the present approach, the results of the RAPD assay in a study by Ozbey *et al. *[23], demonstrated that little genetic heterogeneity exists among the *P. multocida *and *M. haemolytica *isolates. Taylor *et al. *[9] typed 41 *P. multocida *isolates using the RAPD method and observed the similar results, but Dziva *et al. *[14] determined three main clusters in 21 *P. multocida *obtained from pigs. These various results may be due to the use of the different RAPD primers in the different studies. In the present REP-PCR analysis, 131

*P. multocida *isolates typed into three to 18 clusters based on the animal source. In an earlier study in India [22], the REP-PCR analysis results yielded 23 profiles in eight clusters from 67 *P. multocida *strains and the range of the bands was between 350 and 2997 bp that was in agreement with the results of the present study. Gunawardana *et al. *[28] obtained 12–19 bands ranging from 350 to 3600 bp in their study, which shows more bands as compared to this research and also the study by Saxena *et al. *[22], but the range of bands was the same, although in all of the studies the same primer sequence had been used for the amplification. Saxena *et al. *[22] used the purified genomic DNA, which was in agreement with the present study, while Gunawardana *et al. *[28] used the whole-cell lysate PCR for their research. Thus, this could lead to fewer bands in the same range of size in the case of the present approach. Shivachandra *et al. *[20] observed 54 distinct profiles among 66 strains with the band sizes between 200 and 3000 bp and obtained 12 clusters with 80% cutoff. These results were in agreement with the present work, which reflected the apparent polymorphism among the strains based on the amplification of DNA between adjacent repetitive elements.

There was no specific clustering of isolates depending on their geographic or animal host properties, as the isolates with the different geographic or animal origin exhibited cross-clustering. These results were in line with the previous studies. Dziva *et al. *[16] observed cross-clustering among three herds of pigs with the different geographic origin. In addition, Shivachandra *et al. *[20] characterized the avian strains of *P. multocida *and found that despite the phenotypic similarity of the strains, neither the avian host nor the geographical area of the isolation correlated well with the typing profiles. The cross-clustering among the different animal origins represent that *P. multocida *strains can be transmitted between the different species of animals. This finding is supported by a previous study [22]. Additionally, in the present study, isolates from the same species and/or geographical area were located in the different clusters. With the same primer for REP-PCR, Shivachandra *et al. *[20] discussed the same results. These results indicated that Pasteurellosis may be caused by different clones in the same herd or animal. The results based on the RAPD and REP-PCR methods suggested that there is a significant diversity among the isolates with different anatomical locations. If the isolates with different anatomical location (like isolates obtained from Upper respiratory tract (URT) of a healthy animal and the lungs from a diseased animal) had cross-clustering, this diversity could support the hypothesis that *P. multocida *is an opportunistic pathogen. However, an additional investigation is needed to examine the diversity among the strains in the healthy and diseased animals. Taylor *et al. *[19] suggested that the *M. haemolytica *isolates (another genus in pasteurellaceae family) from URT can harbor high diversity, such that only a small portion of strains are associated with the disease. In addition, the isolates associated with the disease may originate in the tonsillar region. Therefore, *M. haemolytica *could be an opportunistic pathogen, too. Thus, the diversity within and concordance between the nasal passage, tonsil and lung warrants additional investigation [19].

In the present study, although both the techniques were able to discriminate between the isolates, the REP-PCR had a slightly higher level of discrimination power than the RAPD analysis and could identify greater diversity within a given group of isolates. Gunawardana *et al. *[28] suggested that the REP-PCR method has a greater level of differentiation than Restriction Endonuclease Analysis (REA), ribotyping, and RAPD. However, further work is justified to confirm this statement or to determine if one method, or a combination of two or more approaches, is more effective in the characterization of *P. multocida *strains.

In conclusion, the results of this research support the previous observation that the REP-PCR method followed by the RAPD assay are the effective molecular tools for epidemiological studies and characterization of *P. multocida*. Furthermore, the results indicated that *P. multocida *isolates have a large amount of genetic heterogeneity and Pasteurellosis may be caused by different clones in the same herd or animal.
